# Blockade of Exosome Release Suppresses Atrial Fibrillation by Alleviating Atrial Fibrosis in Canines With Prolonged Atrial Pacing

**DOI:** 10.3389/fcvm.2021.699175

**Published:** 2021-10-15

**Authors:** Yajun Yao, Shanqing He, Youcheng Wang, Zhen Cao, Dishiwen Liu, Yuntao Fu, Huiyu Chen, Xi Wang, Qingyan Zhao

**Affiliations:** ^1^Department of Cardiology, Renmin Hospital of Wuhan University, Wuhan, China; ^2^Cardiovascular Research Institute, Wuhan University, Wuhan, China; ^3^Hubei Key Laboratory of Cardiology, Wuhan, China

**Keywords:** exosomes, atrial fibrillation, fibrosis, MiR-21-5p, TIMP3, canine

## Abstract

**Background:** Clinical studies have shown that exosomes are associated with atrial fibrillation (AF). However, the roles and underlying mechanisms remain unclear. Hence, this study aimed to investigate the function of exosomes in AF development.

**Methods:** Twenty beagles were randomly divided into the sham group (*n* = 6), the pacing group (*n* = 7), and the pacing + GW4869 group (*n* = 7). The pacing and GW4869 groups underwent rapid atrial pacing (450 beats/min) for 7 days. The GW4869 group received intravenous GW4869 injection (an inhibitor of exosome biogenesis/release, 0.3 mg/kg, once a day) during pacing. Electrophysiological measurements, transmission electron microscopy, nanoparticle tracking analysis, western blotting, RT-PCR, Masson's staining, and immunohistochemistry were performed in this study.

**Results:** Rapid atrial pacing increased the release of plasma and atrial exosomes. GW4869 treatment markedly suppressed AF inducibility and reduced the release of exosomes. After 7 days of pacing, the expression of transforming growth factor-β1 (TGF-β1), collagen I/III, and matrix metalloproteinases was enhanced in the atrium, and the levels of microRNA-21-5p (miR-21-5p) were upregulated in both plasma exosomes and the atrium, while the tissue inhibitor of metalloproteinase 3 (TIMP3), a target of miR-21-5p, showed a lower expression in the atrium. The administration of GW4869 abolished these effects.

**Conclusions:** The blockade of exosome release with GW4869 suppressed AF by alleviating atrial fibrosis in a canine model, which was probably related to profibrotic miR-21-5p enriched in exosomes and its downstream TIMP3/TGF-β1 pathway.

## Introduction

Atrial fibrillation (AF) is the most commonly sustained tachyarrhythmia in the clinic, and its incidence is increasing markedly with the aging population (1). Previous studies have shown that cellular Ca^2+^-handling abnormalities and ectopic/triggered activity led to the initiation of AF, while cardiac structural remodeling provides substrates for the maintenance of AF (2). Fibrosis is the main pathological process of structural remodeling. Although inflammation, oxidative stress, and microRNAs (miRNAs) partly account for it (3), the molecular mechanisms of atrial fibrosis during AF remain to be elucidated.

Exosomes are 40–150-nm small extracellular vesicles secreted by most cells. RNA (including mRNA, miRNA, and other non-coding RNA), proteins, and lipids are selectively incorporated into exosomes and then released into the extracellular space (4). In recent years, growing evidence has shown that exosomes mediate cardiac fibrosis by protein interactions or post-transcriptional translation regulation (5). The renin–angiotensin system is believed to be a major contributor to cardiac fibrosis. Pironti et al. found that heart tissue could release functional angiotensin II type I receptor-enriched exosomes in a mouse model with cardiac pressure overload (6). miRNAs, another major component of exosomes, have also been shown to be involved in the pathogenesis of cardiac fibrosis. Yang et al. identified that cardiomyocyte-derived exosomes containing miR-208a promoted cardiac fibroblast (CF) proliferation and differentiation into myofibroblasts (7). Nevertheless, several studies have confirmed that exosomal miRNAs suppress CF proliferation and antagonize fibrosis progression (8). Exosomes can function differently under different stimulation conditions and microenvironments. What roles exosomes play in the progression of AF remain unknown.

To date, evidence regarding associations between exosomes and AF has mainly focused on clinical research. Some scholars found that plasma exosomes differentially expressed miRNAs between AF and sinus rhythm (9, 10). Bioinformatics analysis showed that these miRNAs and their targets possibly contribute to atrial fibrosis (11). Moreover, it has been demonstrated that, compared with the control group, the epicardial adipose tissue of patients with AF secretes more exosomes, and the expression of pro-inflammatory and pro-fibrotic factors is higher in exosomes (12). In addition, a recent study reported that myofibroblast-derived exosomes reduced the expression of voltage-gated L-type calcium channel subunit α1c (Ca_v_1.2) and increased the vulnerability to AF (13). Based on these studies, we hypothesized that the increased secretion of exosomes was likely to promote AF.

In this study, we aimed to observe the change in exosome release as well as the effect of exosomes on AF inducibility and atrial fibrosis in a canine model of AF. Furthermore, we also tried to investigate the possible mechanism of cardiac fibrosis mediated by exosomes.

## Materials and Methods

This study was approved by the animal studies subcommittee of our institutional review board and was in accordance with the guidelines of the National Institutes of Health for the care and use of laboratory animals.

### Animal Model Preparation

All male beagle canines were maintained under the same conditions in the Animal Experimental Center of Renmin Hospital at Wuhan University. Canines aged 8–12 months with body weights of 8–10 kg were randomly assigned to three groups. The sham group (*n* = 6) received pacemaker implantation under sterile conditions without atrial pacing. The pacing group (*n* = 7) received pacemaker implantation with continuous rapid atrial pacing (450 beats/min) for 7 days. The pacing + GW4869 group (*n* = 7) underwent the same pacing model as the pacing group while administered with a slow intravenous injection (0.3 mg/kg, once a day) of GW4869 (MedChemExpress, USA). Each canine received an intravenous injection of 30 mg/kg pentobarbital sodium before the operation. After successful anesthesia, intubation and ventilation with room air supplemented with oxygen from a respirator (MAO01746, Harvard Apparatus Holliston, USA) and continuous ECG monitoring were performed.

### Cardiac Pacemaker Implantation

Under fluoroscopic guidance, the atrial electrode was implanted in the right atrial appendage for stimulation through the right external jugular vein and then connected to the high-frequency pacemaker placed in the pouch made under the subclavian skin. The pacemaker was programmed to stimulate the right atrium at a frequency of 450 beats/min. After the right atrium showed successful pacing, the electrode was fixed, and the pouch was sutured. Before prolonged pacing started, the canines received post-operative recovery for 3 days, and four million units of penicillin were intramuscularly injected twice a day.

### Electrophysiological Measurements

**Figure 1:**
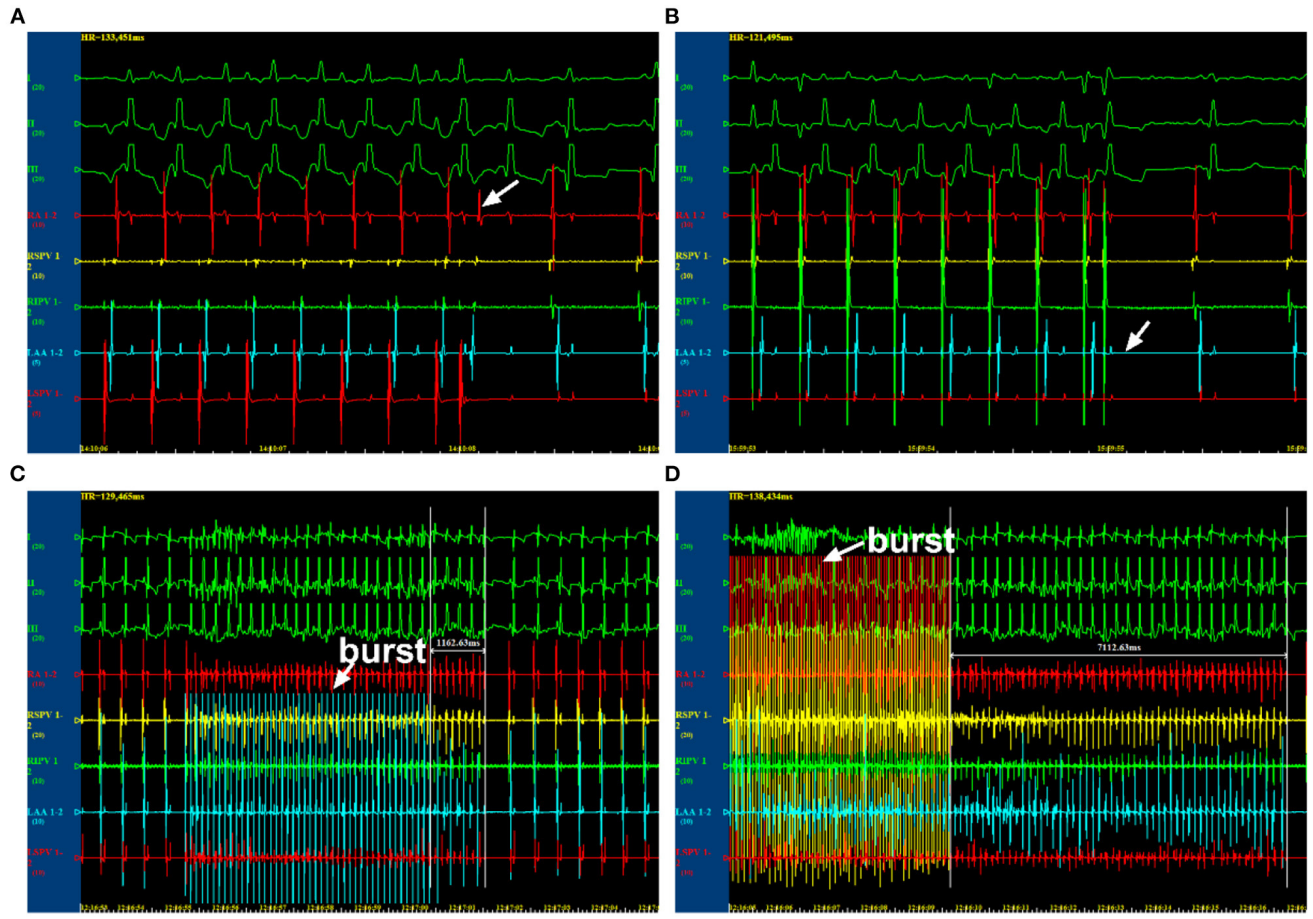
Electrophysiological examination by programmed stimulations. **(A)** The state at which AERP was not reached. **(B)** The state at which AERP was reached. **(C)** The state at which atrial fibrillation (AF) was unable to be induced. **(D)** The state at which AF was induced. AERP, atrial effective refractory period.

Multielectrode catheters were guided to the four pulmonary veins and the left and right atrium. The atrial effective refractory periods (AERPs) were measured using the S_1_S_2_ programmed stimulation protocol (including eight S_1_ regular train stimuli and one S_2_ pre-mature stimulus at twice the pacing threshold intensity). The cycle length (CL) of S_1_ was 250 ms, whereas S_1_S_2_ intervals started as 180 ms and were first decreased by 10 ms and then reduced by 2 ms with each stimulus cycle until the AERP was reached. The longest S1S2 interval without atrial pacing induced by S_2_ pre-mature stimulus was recorded as AERP (Figures 1A,B). The dAERP was calculated by the maximum AERP minus the minimum AERP at all recording sites. The inducibility and duration of AF were assessed using programmed S_1_S_1_ stimulation (a 5-s burst at CL of 120, 100, 75, and 60 ms, three times at every frequency). AF was defined as an irregular atrial rate >500 lasting for more than 5 s (Figures 1C,D). AF inducibility and AF duration were determined by the number of episodes and the maximum duration induced by all bursts of every canine, respectively. Data were recorded by a computerized electrophysiology system (Lead 7000, China). During these operations, canines were supplied with 5 mg/kg pentobarbital sodium to maintain anesthesis every 2 h.

### Histology and Immunohistochemistry

At the end of the experiment, the canines were euthanized with an intravenous injection of excess pentobarbital sodium. The hearts were quickly excised and washed with phosphate-buffered saline (PBS). Then, the atrial tissues were fixed with 4% paraformaldehyde and paraffin-embedded. Deparaffined sections (4 μm in thickness) were stained with Masson's trichrome reagent. The degree of atrial fibrosis was calculated as the percentage of collagen area. For immunohistochemistry staining, the deparaffined sections were subjected to heat-mediated antigen retrieval. The primary antibody anti-CD63 (Sanying Biotechnology, China) was used to incubate atrial samples, followed by secondary antibody HRP-labeled goat anti-rabbit IgG (ASPEN Biotechnology, China). Image-Pro Plus 6.0 software (Media Cybernetics, USA) was used to analyze images. Three visual fields of the right atrium at ×200 were tested randomly in each sample.

### Exosome Isolation

Blood samples were obtained from the jugular vein and centrifuged at 3,000 rpm for 10 min to remove cellular debris. The supernatants were transferred to fresh tubes and stored at −80°C until analysis. Exosomes were isolated using Total Exosome Isolation Reagent (from serum) (Invitrogen, USA) according to the instructions of the manufacturer. A one-quarter volume of isolation reagent was added to each serum sample, and then the samples were mixed and kept at 4°C for 30 min. Each mixture was centrifuged at 10,000 g for 10 min, and the supernatants were removed. The pellet was resuspended in 100–150 μl of 0.22-μm filtered cold PBS and stored at −80°C for subsequent analysis. The volumes of serum were recorded to calculate the concentration of exosomes.

### Exosome Characterization

Exosomes were characterized by transmission electron microscopy (TEM), nanoparticle tracking analysis (NTA) and western blotting. For TEM, 100 μl of isolated exosomes was fixed in 25% glutaraldehyde overnight, dropped onto a copper net, and stained with 2% phosphotungstic acid oxalate for 2 min. Images were acquired by a transmission electron microscope (HT-7700, Japan). For NTA, briefly, 50 μl of isolated exosomes were washed and diluted to the appropriate concentration with 1X PBS buffer. The volumes and dilution ratios of exosomes were recorded. The size range and particle concentration of the exosomes were examined using a Zeta View instrument (PMX 110, Germany). The expression of exosome marker proteins (CD63, CD81, and TSG101) was determined by western blotting. The antibody information is listed in the western blotting section.

### Real-Time Fluorescent Quantitative PCR

Total RNA was extracted from the isolated exosomes and atrial tissues using TRIpure Total RNA Extraction Reagent (ELK Biotechnology, China) according to the protocols of the manufacturers. The relative levels of miR-21-5p and U6 were detected by Stem-Loop RT-qPCR using an Enturbo™ SYBR Green PCR Supermix Kit (ELK Biotechnology, China) with a StepOne™ Real-Time PCR instrument (Life Technology, USA). The data were analyzed by 2^−ΔΔCT^ method.

### Western Blotting

Total proteins were extracted from atrial samples using radioimmunoprecipitation assay buffer plus phosphoprotease inhibitors (ASPEN Biotechnology, China). The same amount (40 μg) of extracted protein was separated by electrophoresis in SDS-PAGE gels and transferred to polyvinylidene difluoride membranes. Membranes were blocked with 5% blocking buffer for 1 h at room temperature and incubated overnight at 4°C with primary antibodies against CD63 (Biorbyt, England), CD81 (Abcam, USA), TSG101 (Sigma-Aldrich, Germany), Rab27a (Sanying Biotechnology, China), collagen I (Novusbio, USA), collagen III (Abcam, USA), matrix metalloproteinase (MMP)-2 (Bioss, USA), MMP-9 (Bioss, USA), tissue inhibitor of metalloproteinase 3(TIMP3) (Lsbio, USA), and transforming growth factor-β1 (TGF-β1) (Sanying Biotechnology, China). The membranes were washed three times with tris-buffered saline with 0.1% Tween® 20 and then incubated with horseradish peroxidase-conjugated anti-rabbit secondary antibody (ASPEN Biotechnology, China) for 1 h at room temperature. The blots were exposed with an ECL Detection Kit (ASPEN Biotechnology, China). The expression levels of the proteins were determined and normalized to the relative intensity of GAPDH using image analyzer software (AlphaEase FC, USA).

### Statistical Analysis

All data are expressed as mean ± standard deviation. Two-sample independent Student's *t*-test was performed to compare the means of two groups. ANOVA, followed by Newman–Keuls tests, was used to compare the mean values of continuous variables among multiple groups, and any significant differences were further analyzed using the Tukey–Kramer test. All statistical tests were two-sided, and a probability value <0.05 was considered statistically significant. All data were analyzed using GraphPad Prism 8 (GraphPad, USA).

## Results

### Prolonged Rapid Atrial Pacing Increased the Release of Plasma Exosomes

**Figure 2:**
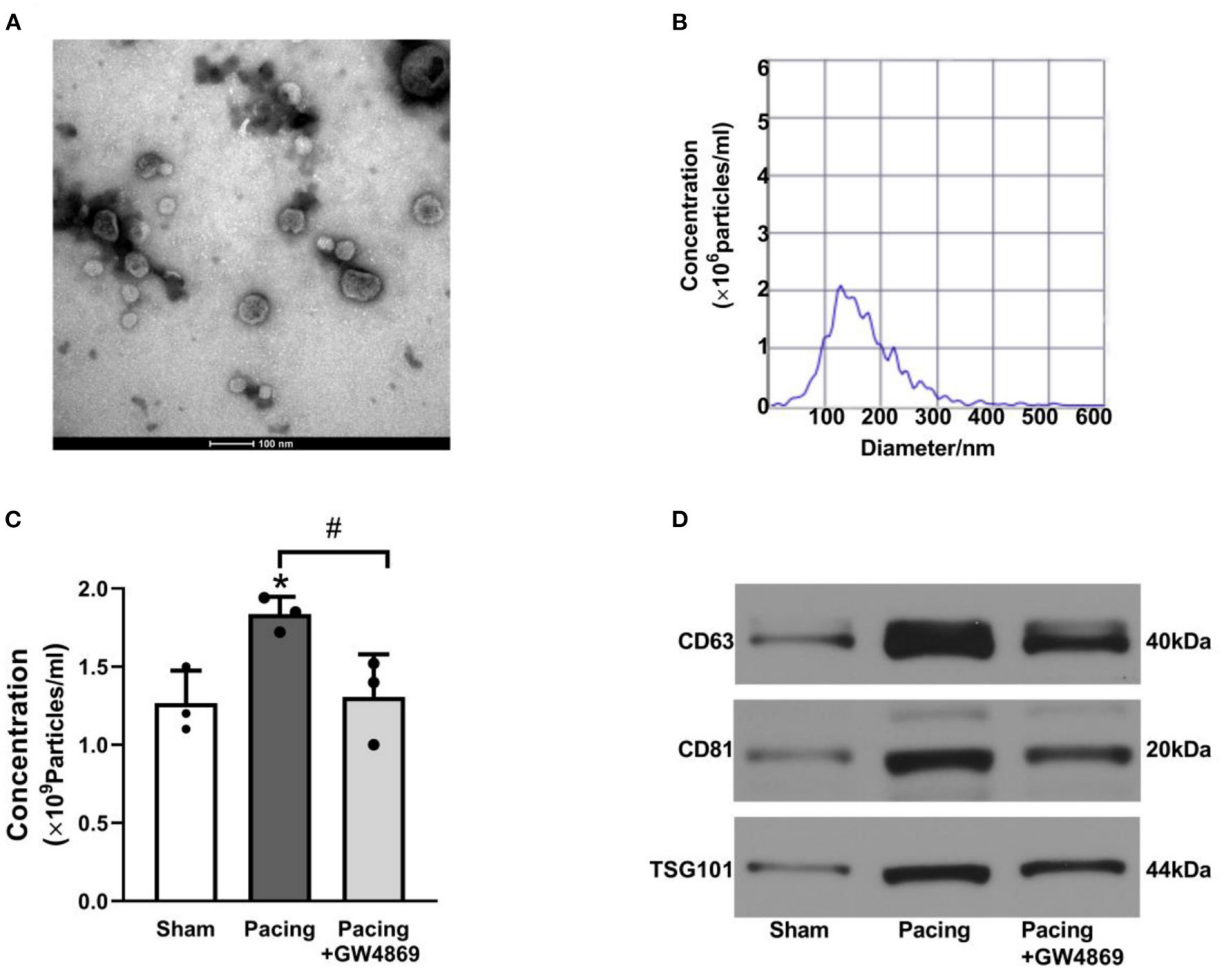
Characteristics of plasma exosomes in a canine model of AF. **(A)** Representative electron microscopy image of exosomes isolated from plasma (*n* = 3, scale bar, 100 nm). **(B)** Representative NTA picture of the exosome size range and concentration (*n* = 3; dilution ratio, 1:500). **(C)** The concentration of plasma exosomes according to NTA analysis and plasma volume. **(D)** Representative western blotting images of the exosome markers CD63, CD81, and TSG101. *P < 0.05 vs. the sham group. ^#^*P* < 0.05 vs. the pacing group. AF, atrial fibrillation; NTA, nanoparticle tracking analysis.

As shown in Figure 2, TEM, NTA, and western blotting were used to characterize the exosomes isolated from plasma. TEM showed that isolated exosomes were extracellular vesicles with a diameter of ~30–150 nm (Figure 2A). NTA further measured the size distribution and approximate concentration of the vesicles. The size of the vesicles ranged between 30 and 300 nm, most of which were 100–150 nm in diameter (Figure 2B). Plasma exosome concentrations were calculated according to NTA results and plasma volume. After rapid atrial pacing, the concentration of plasma exosomes rose to (1.84 ± 0.11) × 10^9^ vesicles/ml from (1.27 ± 0.21) × 10^9^ vesicles/ml, which was decreased to (1.31 ± 0.27) × 10^9^ vesicles/ml by GW4869 treatment (both *P* < 0.05; Figure 2C). Western blotting showed that the vesicles expressed the exosome markers CD63, CD81, and TSG101 (Figure 2D). The results suggested that prolonged rapid atrial pacing increases the release of plasma exosomes, which was dampened by GW4869 treatment.

### Prolonged Rapid Atrial Pacing Increased the Release of Atrial Exosomes

**Figure 3:**
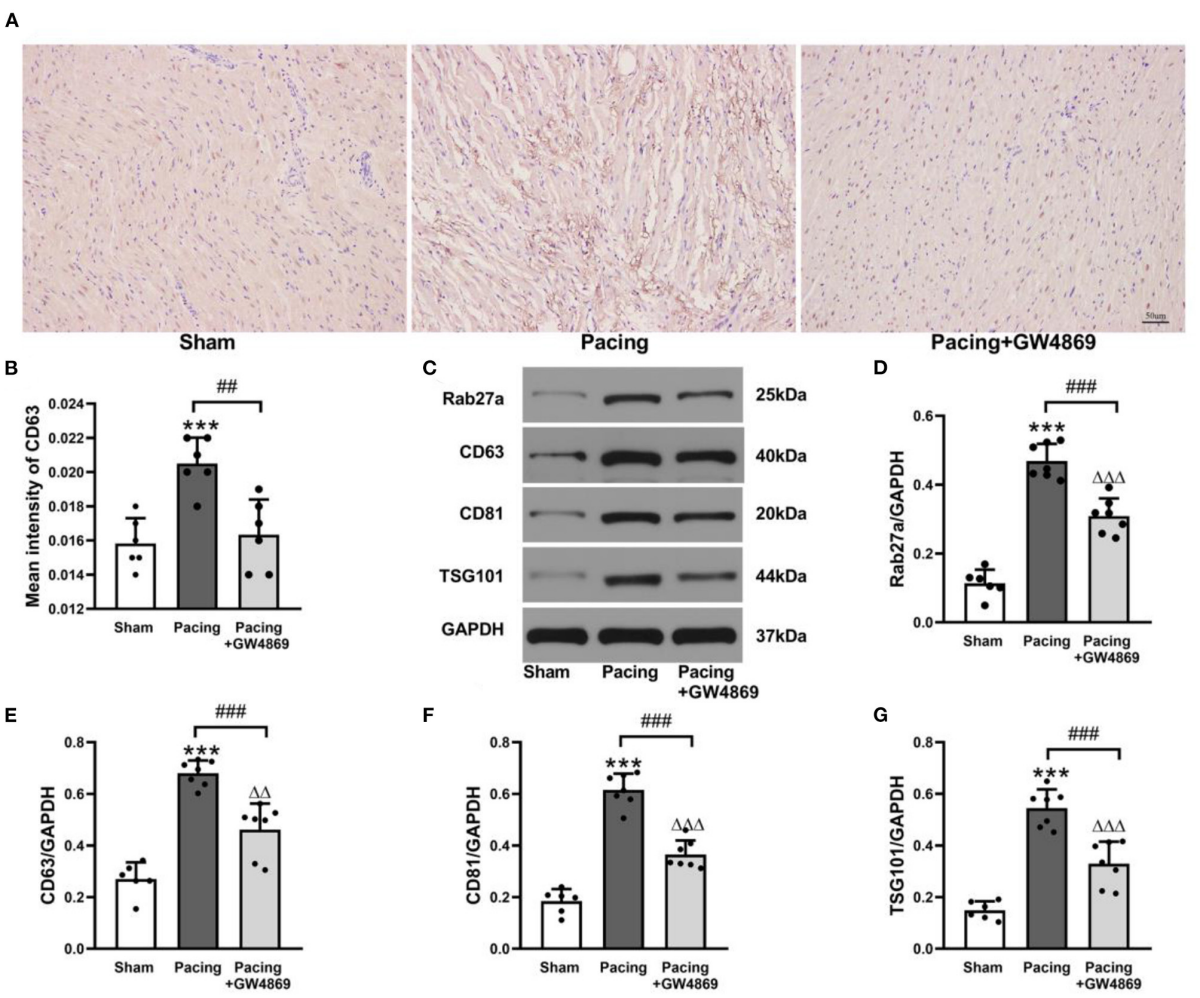
Analysis of exosomal marker proteins in the atria from a canine AF model. **(A,B)** Immunohistochemistry staining of CD63 and mean values of CD63 intensity in the atria (*n* = 6, ×200). **(C)** Representative western blotting images of Rab27a, CD63, CD81, and TSG101 in the atria (*n* = 6 for the sham group and *n* = 7 for the pacing and GW4869 groups). **(D–G)** The mean expression levels of Rab27a, CD63, CD81, and TSG101 in the atria (*n* = 6 for the sham group and *n* = 7 for the pacing and GW4869 groups). ***P < 0.001 vs. the sham group. ^*##*^*P* < 0.01 vs. the pacing group, ^###^*P* < 0.001 vs. the pacing group. ^ΔΔ^*P* < 0.01, ^ΔΔΔ^*P* < 0.001 vs. the sham group. AF, atrial fibrillation.

As shown in Figure 3, immunohistochemistry staining and western blotting were used to analyze the expression of exosome marker proteins in the atria. Immunohistochemistry staining showed that the mean density of the exosome marker CD63 in the pacing group was distinctly higher than that in the sham group and the pacing + GW4869 group (both *P* < 0.01; Figures 3A,B). To further investigate the change in atrial exosome secretion, Rab27a (an important membrane protein associated with exosome secretion) and exosome markers, such as CD63, CD81, and TSG101, were examined by western blotting. Compared to the sham group, the levels of Rab27a, CD63, CD81, and TSG101 in the atrium were greatly higher in the pacing group (Rab27a: 0.47 ± 0.05 vs. 0.11 ± 0.04; CD63: 0.68 ± 0.05 vs. 0.27 ± 0.06; CD81: 0.62 ± 0.06 vs. 0.18 ± 0.05; TSG101: 0.54 ± 0.07 vs. 0.15 ± 0.04; all *P* < 0.001) and were reduced in the pacing + GW4869 group (all *P* < 0.001). The expression levels of exosome marker proteins remained different between the sham group and the pacing + GW4869 group (all *P* < 0.01; Figures 3C–G). These results indirectly showed that prolonged rapid atrial pacing could increase the release of atrial exosomes, which were reduced by GW4869 treatment.

### Inhibition of Exosome Release Lightened AERP Shortening Caused by Prolonged Rapid Atrial Pacing

**Figure 4:**
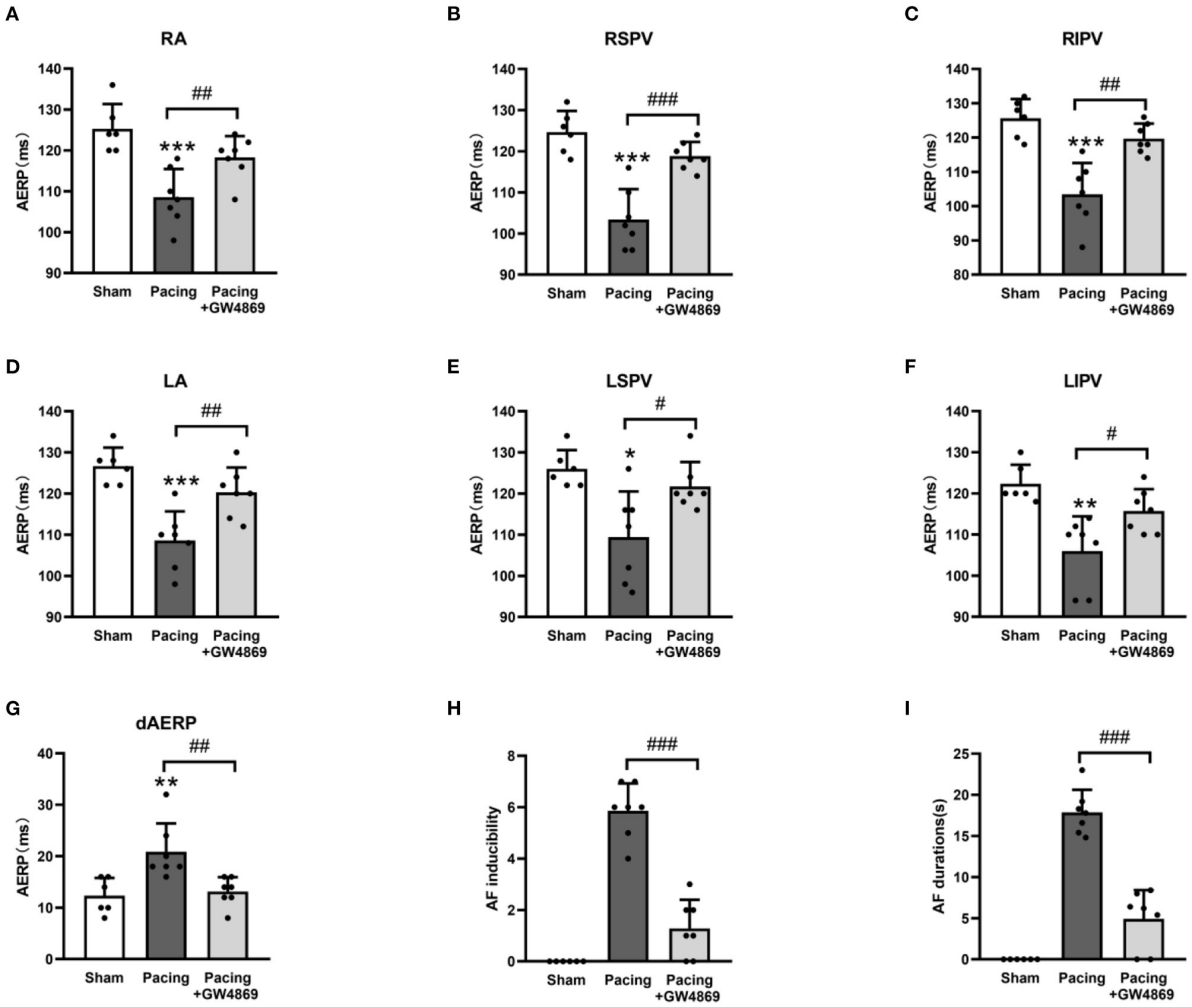
Electrical remodeling in a canine model of AF. **(A–F)** Differences in AERPs at the RA, RSPV, RIPV, LA, LSPV, and LIPV (*n* = 6 for the sham group and *n* = 7 for the pacing and GW4869 groups). **(G)** Difference in the dAERP (*n* = 6 for the sham group and *n* = 7 for the pacing and GW4869 groups). **(H)** Difference in AF inducibility, shown by the number of episodes (*n* = 6 for the sham group and *n* = 7 for the pacing and GW4869 groups). **(I)** Difference in mean AF durations (*n* = 6 for the sham group and *n* = 7 for the pacing and GW4869 groups). *P < 0.05, **P < 0.01, ***P < 0.001 vs. the sham group. ^#^*P* < 0.05, ^##^*P* < 0.01, ^###^*P* < 0.001 vs. the pacing group. AERP, atrial effective refractory period; dAERP, dispersion of atrial effective refractory period; RA, right atrium; RSPV, right superior pulmonary vein; RIPV, right inferior pulmonary vein; LA, left atrium; LSPV, left superior pulmonary vein; LIPV, left inferior pulmonary vein; AF, atrial fibrillation.

To evaluate the effect of exosome inhibition on cardiac electrophysiology, AERPs at different sites were recorded. As shown in Figure 4, compared with the sham group, AERPs at the six recorded sites were significantly decreased in the pacing group (all *P* < 0.05), which were mitigated by the administration of GW4869 (all *P* < 0.05; Figures 4A–F)—for example, the AERP at RA was 125 ± 6 ms in the sham group, 109 ± 7 ms in the pacing group (*P* < 0.001 vs. the sham group), and 118 ± 5 ms in the pacing + GW4869 group (*P* < 0.01 vs. the pacing group). In addition, the dAERP was markedly increased in the pacing group and was reduced in the pacing + GW4869 group (both *P* < 0.01) (Figure 4G). There were no significant differences in the AERP and dAERP between the sham group and pacing + GW4869 group.

### Inhibition of Exosome Release Dramatically Reduced AF Inducibility and Persistence After Prolonged Rapid Atrial Pacing

The inducibility and duration of AF were assessed through S1S1 programmed stimulus. AF inducibility was increased in the pacing group and was dramatically lessened by GW4869 treatment (*P* < 0.001; Figure 4H). In addition, the prolonged AF durations after rapid atrial pacing were significantly shortened by GW4869 (4.91 ± 3. 51 vs. 17.87 ± 2. 75 s, *P* < 0.001; Figure 4I). These results showed that inhibition of exosome release exerted a potential inhibitory effect on the inducibility and persistence of AF.

### Inhibition of Exosome Release Suppressed AF by Ameliorating Atrial Fibrosis

**Figure 5:**
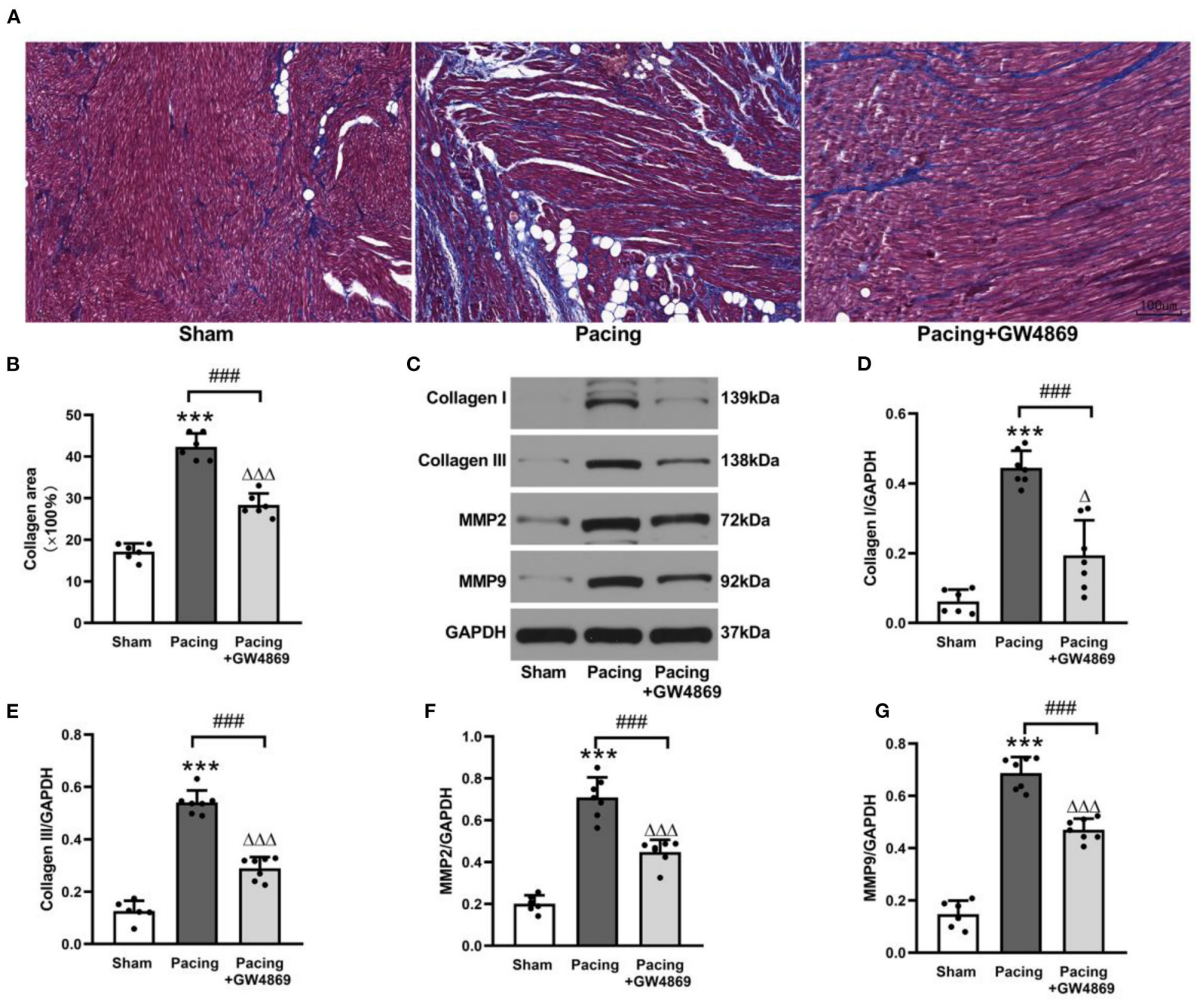
Structural remodeling in a canine model of AF. **(A,B)** Masson's trichrome staining of the atria and mean values of collagen area in the atria (*n* = 6, ×200). **(C–G)** Representative Western blotting images and the mean expression levels of collagen I, collagen III, MMP-2, and MMP-9 in the atria (*n* = 6 for the sham group and *n* = 7 for the pacing and GW4869 groups). ***P < 0.001 vs. the sham group. ^###^*P* < 0.001 vs. the pacing group. ^Δ^*P* < 0.05, ^ΔΔΔ^*P* < 0.001 vs. the sham group. MMP-2, matrix metalloproteinase-2; MMP-9, matrix metalloproteinase-9; AF, atrial fibrillation.

As shown in Figure 5, the degree of atrial fibrosis was evaluated by Masson's trichrome staining and western blotting. Compared with that in the sham group, collagen deposition in the atrium was observably increased in the pacing group (42.33 ± 3.20% vs. 17.17 ± 1.94%, *P* < 0.001) and was reduced by GW4869 treatment (28.33 ± 2.81% vs. 42.33 ± 3.20%, *P* < 0.001) (Figures 5A,B). Consistent with this result, western blotting showed that the expression of collagen I, collagen III, MMP-2, and MMP-9 in the atria was greatly enhanced in the pacing group (all *P* < 0.001) and repressed in the pacing + GW4869 group (all *P* < 0.001; Figures 5C–G)—for example, after rapid atrial pacing, the relative level of collagen I was increased from 0.06 ± 0.03 to 0.44 ± 0.05, and GW4869 treatment restored it to 0.19 ± 0.10 (both *P* < 0.001). These data indicated that the increased release of exosomes caused by prolonged rapid atrial pacing primarily played a pro-fibrotic role; therefore, inhibition of exosome release ameliorated atrial fibrosis in a canine model of AF.

### Exosomes Mediated Atrial Fibrosis Through Exosomal Pro-fibrotic miR-21-5p and Its Downstream Pathway

**Figure 6:**
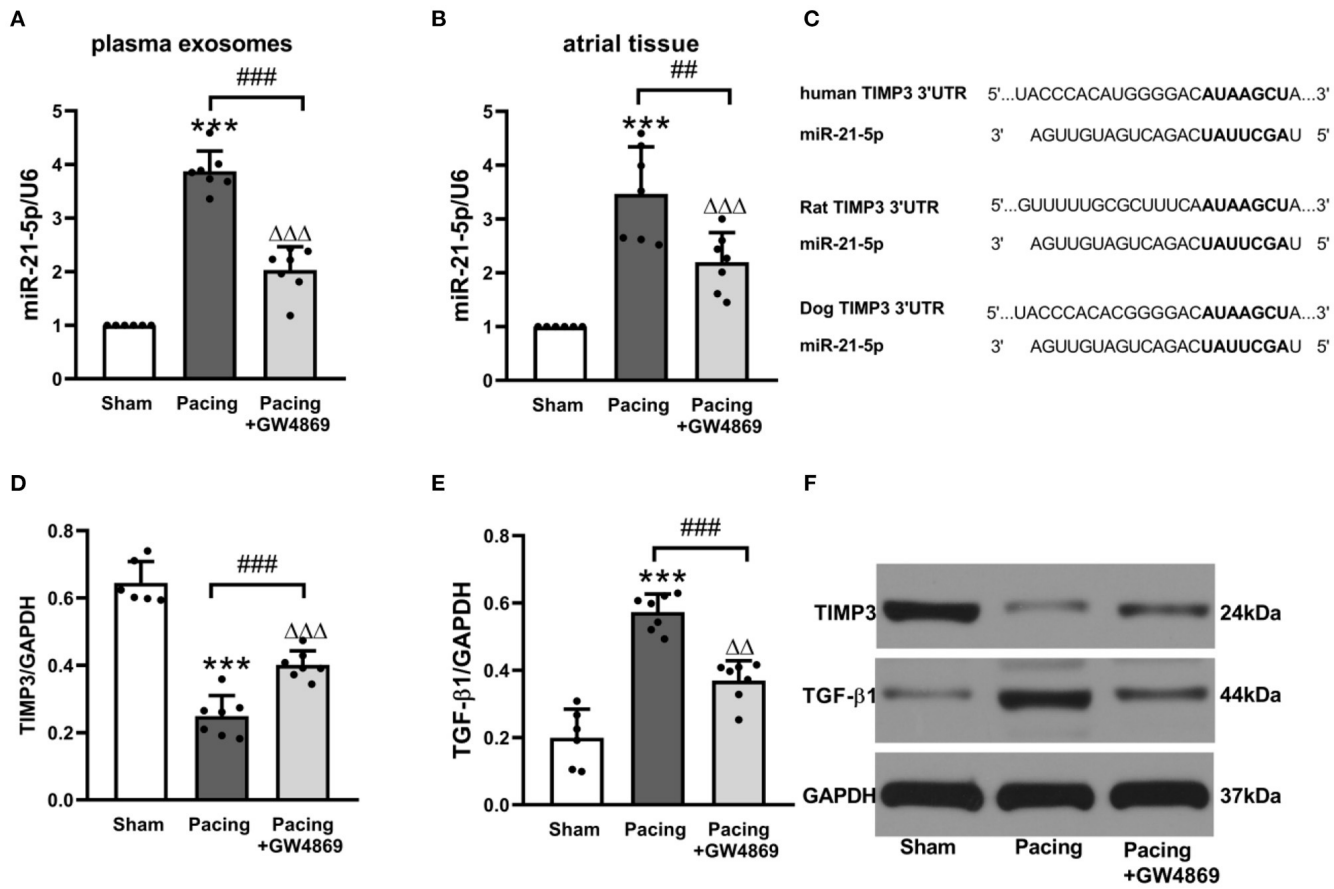
Possible mechanism of atrial fibrosis mediated by exosomes. **(A,B)** Relative levels of miR-21-5p and U6 in plasma exosomes and atrial tissue (*n* = 6 for the sham group and *n* = 7 for the pacing and GW4869 groups). **(C)** Predicted miR-21-5p target sequences in the 3′UTR of TIMP3 among humans, rats, and dogs. **(D–F)** Representative Western blotting images and the mean expression levels of TIMP3 and TGF-β1 in the atria (*n* = 6 for the sham group and *n* = 7 for the pacing and GW4869 groups). ***P < 0.001 vs. the sham group. ^##^*P* < 0.01, ^###^*P* < 0.001 vs. the pacing group.^ΔΔ^*P* < 0.01, ^ΔΔΔ^*P* < 0.001 vs. the sham group. miR-21-5p, microRNA-21-5p; TIMP3, tissue inhibitor of metalloproteinase 3; TGF-β1, transforming growth factor-β1; AF, atrial fibrillation.

To further explore the relationship between exosomes and atrial fibrosis, qRT-PCR was used to verify pro-fibrotic miRNA expression in plasma exosomes and atrial tissues. As shown in Figure 6, compared with the sham group, plasma exosomal miR-21-5p was significantly increased in the pacing group (*P* < 0.001), and administration of GW4869 reversed this result (*P* < 0.001). Moreover, the expression pattern in atrial tissue was consistent with that in plasma exosomes (Figures 6A,B). Bioinformatics analyses indicated that miR-21-5p targeted TIMP3, which participated in fibrosis *via* the TIMP3/TGF-β pathway (Figure 6C). As anticipated, the expression level of TIMP3 in the pacing group was lower than that in the sham group (0.25 ± 0.06 vs. 0.64 ± 0.06, *P* < 0.001) and in the pacing + GW4869 group (0.25 ± 0.06 vs. 0.40 ± 0.04, *P* < 0.001). The expression of TGF-β1 was inversely correlated with TIMP3 (Figures 6D–F). These data illustrated that exosome-mediated atrial fibrosis partly resulted from exosomal pro-fibrotic miR-21-5p and its downstream TIMP3/TGF-β1 pathway in a canine model of AF.

## Discussion

This study was the first attempt to explore the effect of exosomes on AF inducibility and maintenance in a canine model of rapid atrial pacing. We provided novel evidence as follows: (1) rapid atrial pacing increased the release of plasma and atrial exosomes in canines; (2) increased exosomes mainly played a pro-fibrotic role in AF; thus, blockade of exosome release with GW4869 suppressed AF by alleviating atrial fibrosis; and (3) this pro-fibrotic effect of exosomes partly resulted from miR-21-5p enrichment in exosomes and its downstream pathway of TIMP3/TGF-β1.

Exosomes, as important mediators of cellular signal transduction and intercellular communication, have been demonstrated to participate in some cardiovascular diseases, such as coronary atherosclerosis, myocardial infarction, and heart failure (14, 15). GW4869, a non-competitive neutral sphingolipase (N-SMase) inhibitor, has been used as an inhibitor of exosome synthesis and release in some studies (16–18). A recent study verified that coronary artery ligation increased the local release of large and small extracellular vesicles in a murine model of MI (19). Blocking the release of exosomes with GW4869 alleviated the lipopolysaccharide-induced myocardial inflammatory response and improved the cardiac function (20).

As the most commonly sustained arrhythmia, the mechanism and treatment of AF remain suboptimal. Several studies have shown that miRNAs in exosomes are more stable and sensitive than those in blood and thus have a potential role as clinical biomarkers for AF (9). Similarly, due to the vesicular structure, the paracrine mode of action, and fusion with recipient cells, exosomes can be modified specifically to carry specific substances to recipient cells for AF treatment (21). Nevertheless, little is known regarding the association between exosomes and the pathological processes of AF in animal models. In the present study, we established an AF model by rapid atrial pacing in canines. The concentration analysis of plasma exosomes showed that exosome release was increased in the pacing group. Meanwhile, western blotting confirmed the higher expression of vesicle secretion-associated protein Rab27a and exosome marker proteins CD63, CD81, and TSG101 in the paced atria. The release of exosomes was increased after prolonged rapid atrial pacing. The shortening of the AERPs in specialized heart tissues caused differential excitability and conductivity at different heart sites, contributing to AF inducibility and persistence after rapid atrial pacing. By electrophysiological measurements, we observed that shortened AERPs and increased AF inducibility after rapid atrial pacing were alleviated by GW4869 treatment. Treatment with GW4869 also significantly shortened the AF durations. These results indicated that increased exosomes probably played a vital role in the development of AF. The changes in AERPs could be related to some ion channel-related miRNAs contained in exosomes. A previous study also showed that Ang II-treated cardiac fibroblast-derived exosomes contained miR-21-3p, which could regulate the expression of Cav1.2 and contribute to the development of a susceptible substrate for atrial fibrillation (13). However, we did not further explore the mechanism by which inhibition of exosome release directly changed AERPs.

Atrial fibrosis is an important contributor to AF maintenance, which is not only related to AF mechanisms but also increases the risk of complications and therapeutic failure. It involves a series of complicated processes, including the accumulation of extracellular matrix proteins and the imbalance of enzyme activation and inactivation (22). Previous studies have shown that exosomes participate in fibrosis progression. However, most studies were carried out *in vitro*, which was not enough to justify the overall effect of exosomes on fibrosis and provide insights for clinical transformation. Moreover, the function of exosomes depends strongly on stimulation conditions and microenvironments—for example, Działo et al. found that Wnt5a-enriched exosomes activated the ERK1/2 and JNK pathways, induced the production of IL-6, and promoted fibrosis (23). Wang et al. showed that miR-107 in vascular endothelial cell-derived exosomes could alleviate fibrosis through the HIF-1α/Notch1/PDGFRβ/YAP1/Twist1 pathway (24). Thus, we investigated the impact of exosomes on atrial fibrosis in a canine model of AF. Our results showed that the exosomes increased by rapid atrial pacing markedly exacerbated collagen deposition in the atria and elevated the expression of collagen I, collagen III, MMP-2, and MMP-9, which could be reversed by the administration of GW4869. These results suggested that the enhanced release of exosomes primarily played a pro-fibrotic role in a canine model of AF. Inhibition of exosome release likely provided a novel treatment method for atrial fibrillation by mitigating atrial fibrosis.

Proceeding with this study, we further explored the mechanism of exosomes in pro-fibrotic effects. On account of the inclusion of lipid bilayers, miRNAs in exosomes are generally thought to stay biologically stable (25). Some scholars even consider exosomes to be the main form of miRNAs present in serum. Moreover, abundant studies have unveiled the critical impact that miRNAs exert on the activation of fibrosis signals (26). These findings guided us to investigate the role of miRNAs enriched in exosomes in facilitating atrial fibrosis. Emerging evidence has suggested a role for miR-21 in the development of cardiac fibrosis (27, 28). More importantly, a recent study uncovered that the level of circulating miR-21 was relevant to the echocardiographic parameters of atrial remodeling and prediction of AF (29). Therefore, we sought to validate the expression of miR-21-5p in plasma exosomes and atrial tissue. The data showed that rapid atrial pacing elevated the miR-21-5p expression levels in both plasma exosomes and the atrium. GW4869 treatment antagonized these effects. Thus, we speculated that miR-21-5p enriched in exosomes was related to atrial fibrosis in a canine model of AF.

Subsequently, bioinformatics analysis was performed by TargetScan 7.2 to search the target genes of miR-21-5p. Among the target genes with high correlation, TIMP3 has been demonstrated to be involved in fibrosis progression in an MMP-dependent or MMP-independent manner (30). Loss of TIMP3 increased myocardial fibrosis and elevated the expression of MMP2 and MMP9 to a greater extent in iron-overloaded mice (31). TGF-β plays a central role in cardiac fibrosis and CF function. Another study showed that TIMP3 could modulate the TGF-β expression in Ang II-treated CFs (32). In our study, we explored whether higher levels of exosomal miR-21-5p exacerbated TIMP3 degradation and increased TGF-β1 expression. As expected, the pacing group showed a lower TIMP3 expression and a higher TGF-β1 expression, which were reversed by GW4869 treatment. Moreover, a recent study also showed that a miR-21-5p antagomir could repress myocardial fibrosis by targeting TIMP3 after myocardial infarction (33), which provided an important foundation for our research. These results indicated that the increased release of exosomes induced by prolonged rapid atrial pacing probably promoted atrial fibrosis through the miR-21-5p/TIMP3/TGF-β1 signaling pathway.

## Conclusions

We demonstrated for the first time in a canine model of AF that prolonged rapid atrial pacing increased exosome release and that the blockade of exosome release with GW4869 suppressed AF by alleviating atrial fibrosis, which was related to pro-fibrotic miR-21-5p enriched in exosomes and its downstream TIMP3/TGF-β1 signaling pathway. This study provides a new insight into the mechanism underlying AF maintenance.

### Limitations

In our experiment, we confirmed that GW4869 treatment reduces the release of plasma and atrial exosomes and suppresses AF by alleviating atrial fibrosis. However, the quantitation standard of exosomes has still not reached a consensus in recent years. We assessed the release of exosomes in plasma by NTA analysis, while that in the atrium was assessed by exosome marker protein expression. They were not necessarily perfectly correlated with the exosome number. In addition, we failed to evaluate the precise pharmacokinetics of GW4869 and its potential adverse effects on other organ systems. Finally, we only provided a possible mechanism by which exosomes participate in atrial fibrillation by regulating atrial fibrosis. Whether some other miRNAs exist in exosomes induced by rapid atrial pacing and how they function in AF remains unknown. The miRNA sequencing analysis and specific mechanism still need further study.

## Data Availability Statement

The original contributions presented in the study are included in the article/supplementary material, further inquiries can be directed to the corresponding author.

## Ethics Statement

The animal study was reviewed and approved by Laboratory Animal Welfare and Ethics Committee of Renmin Hospital of Wuhan University.

## Author Contributions

YY and QZ conceived and designed the research. YW, YY, and SH performed the experiments. SH, ZC, and DL analyzed the experiment results. YY drafted and edited the manuscript. YF, HC, and XW revised the manuscript. All authors contributed to the article and approved the submitted version.

## Funding

This work was supported by the National Natural Science Foundation of China (No. 81970277 to QZ).

## Conflict of Interest

The authors declare that the research was conducted in the absence of any commercial or financial relationships that could be construed as a potential conflict of interest.

## Publisher's Note

All claims expressed in this article are solely those of the authors and do not necessarily represent those of their affiliated organizations, or those of the publisher, the editors and the reviewers. Any product that may be evaluated in this article, or claim that may be made by its manufacturer, is not guaranteed or endorsed by the publisher.
